# Energy-dependent cortical injury thresholds in high-frequency transcortical electrical stimulation: a biophysical study in a rat model

**DOI:** 10.1007/s00221-026-07347-1

**Published:** 2026-06-26

**Authors:** Ridzky Firmansyah Hardian, Kunihiko Kodama, Kohei Kanaya, Tetsuya Goto, Kazuhiro Hongo, Tetsuyoshi Horiuchi

**Affiliations:** 1 Department of Neurosurgery, Shinshu University School of Medicine, Matsumoto, Nagano Japan; 2 Department of Neurosurgery, Japanese Red Cross Iiyama Hospital, Iiyama, Nagano, Japan; 3 Department of Neurosurgery, Suwa Red Cross Hospital, Suwa, Nagano Japan; 4 Department of Neurosurgery, Ina Central Hospital, Ina, Nagano Japan; 5 Department of Physiology, Faculty of Medicine, Diponegoro University, Central Java, Indonesia; 6 Department of Neurosurgery, Faculty of Medicine, Diponegoro University, Central Java, Indonesia

**Keywords:** Transcortical stimulation, Cortical lesion depth, Biophysics, Resistive heating, Rat model

## Abstract

The biophysical determinants of cortical tissue injury during brief, high-frequency transcortical electrical stimulation remain incompletely understood. Traditional safety criteria derived from chronic, low-frequency paradigms emphasize total charge, but whether charge or stimulation energy is the primary predictor of injury under high-frequency conditions—such as those used for intraoperative motor evoked potential (MEP) monitoring—is unclear. Thirty-two Sprague-Dawley rats (8 groups of 4) received monophasic anodal transcortical pulse trains with varying current, pulse duration, repetition number, and interstimulation interval. Maximum cortical lesion depth was measured histologically, and the contributions of total charge (Q = I × t) and relative stimulation energy (W ∝ I^2^t) were dissociated through controlled group comparisons and multiple linear regression. Higher stimulation current (*p* = 0.04) and greater repetition number (*p* = 0.005) significantly increased lesion depth. Multiple regression identified total stimulation energy as the only significant independent predictor (*p* = 0.008), whereas total charge was not (*p* = 0.677). With equivalent total charge, higher-energy stimulation tended to produce deeper lesions (209 ± 67 vs. 125 ± 101 μm, *p* = 0.25); when total energy was equalized despite a 2.5-fold difference in total charge, no detectable difference in lesion depth was observed (*p* = 1.0). Under high-frequency conditions, cortical injury depth appears to be more strongly associated with total energy deposition than with total charge alone, consistent with biophysical mechanisms involving resistive heating and electrolytic processes at the electrode–tissue interface. These findings provide a mechanistic framework for understanding electrical tissue injury under MEP-relevant stimulation regimes.

## Introduction

Intraoperative motor evoked potential (MEP) monitoring with transcortical stimulation is widely used to preserve motor pathways during brain surgery (Taniguchi et al. 1993; Krieg et al. 2012; Ichikawa et al. 2010). The technique relies on brief trains of high-frequency pulses (typically several hundred Hz) delivered at intensities often exceeding those used in chronic cortical stimulation paradigms (Pechstein et al. 1996; Oinuma et al. 2007). Despite the widespread clinical use of this stimulation regime, the biophysical determinants of cortical tissue injury under such conditions remain poorly defined.

Traditional safety criteria for cortical stimulation, including Shannon’s model, emphasize charge density per phase and total charge density as primary predictors of neural injury (Shannon 1992; Cogan et al. 2016; Yuen et al. 1981; McCreery et al. 1990; Gordon et al. 1990; MacDonald 2002). However, these criteria were derived from chronic, low-frequency paradigms in which stimulation was delivered continuously over hours to days (Yuen et al. 1981; McCreery et al. 1990; Harnack et al. 2004). The brief, high-frequency, repetitive trains used for intraoperative MEP differ fundamentally from these conditions in their temporal pattern of energy delivery and in the instantaneous power dissipated in tissue. Whether charge-based safety criteria adequately capture the biophysical relationship between stimulation parameters and the resulting volumetric tissue damage in this regime remains an open question.

In the present study, we provide a mechanistic framework by evaluating the relative contributions of electrical charge (Q = I × t) and stimulation energy (W ∝ I^2^t) to the depth of cortical lesions in a rat model under MEP-relevant high-frequency stimulation. By experimentally dissociating these two parameters—comparing groups with matched total charge but different total energy, and vice versa—we sought to identify the primary biophysical driver of tissue injury and to clarify the underlying physical mechanisms, including resistive heating and anodic electrolysis.

## Methods

Thirty-two 8- to 12-week-old male Sprague-Dawley rats (weight 350–450 g) were used for the experiments. The rats were provided by the vivarium of Shinshu University School of Medicine. The animal experiments were conducted in accordance with the guidelines for the care and use of laboratory animals established by Shinshu University’s Ethical Review Board. The rat experiment was approved by the Ethical Committee of Shinshu University (approval no. 200024). All procedures were designed in accordance with the 3R principles (Replacement, Reduction, and Refinement) to minimize the number of animals used and to reduce suffering.

**Fig. 1:**
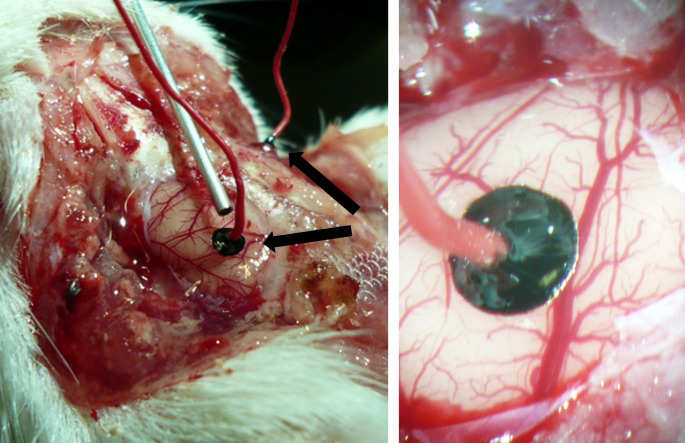
Platinum electrodes (arrows) with a diameter of 2 mm were placed on the bilateral cerebral cortex of the rat (left). The electrode attachment to the cerebral cortex was shown in a magnified photo (right)

After induction of general anesthesia with intraperitoneal pentobarbital (60 mg/kg), all efforts were made to minimize pain and distress. The head was fixed to a rat head-fixation device. Bilateral frontal craniectomies with a diameter of 10 mm were performed, and the dura mater was exposed. The bilateral dura mater was incised circumferentially to expose the cerebral cortex. The arachnoid membrane and the cerebral cortex were kept intact. A pair of platinum electrodes with a diameter of 2 mm (Unique Medical Co. Ltd, Japan) were placed on the cerebral cortex bilaterally (Fig. 1).

Anodal monopolar high-frequency monophasic stimulation, which is a standard MEP stimulation method (Szelényi et al. 2007), was used to deliver electrical stimulation to the cerebral cortex. The placement of the stimulating electrode ensured that stimulation was delivered through the uninjured cortex. Neuropack Σ^®^ (Nihon Kohden, Japan) with a constant-current stimulator was used as a stimulation machine. A five-pulse train stimulation with a pulse frequency of 500 Hz, an interstimulation interval (ISI) of 2 ms, a current of 20 mA or 50 mA, a duration of 0.05 ms, 0.2 ms, or 0.5 ms, an interval between each stimulation session of 1–10 s, and stimulus repetition of 0 (control), 10x, 100x, or 625x was delivered. This stimulation protocol resulted in 8 groups of rats (4 rats per group) with various combinations of stimulation parameters (Table 1).

**Table 1:** Stimulation parameter for each group of the experiments

Rats group	Current (mA)	Duration (ms)	Interstimulation interval (s)	Stimulation number (*n*)	Total charge (µC)	Relative energy (I^2^t, arbitrary units)	Mean lesion depth (µm)
1	50	0.2	1	0	0	0	0
2	50	0.2	1	10	500	25	0
3	50	0.2	1	100	5000	250	209 ± 67
4	20	0.2	1	100	2000	40	97 ± 33
5	50	0.05	1	100	1250	62.5	157 ± 141
6	50	0.2	10	100	5000	250	183 ± 36
7	20	0.2	1	625	12,500	250	211 ± 59
8	20	0.5	1	100	5000	100	125 ± 101

Eight experimental groups were designed to evaluate the following comparisons: (1) the effect of stimulation number (groups 1–3 at 50 mA, and groups 4 and 7 at 20 mA); (2) the effect of stimulation current (groups 3 vs. 4, 50 mA vs. 20 mA, 100 repetitions); (3) the effect of stimulation duration (groups 3 vs. 5 at 50 mA, and groups 4 vs. 8 at 20 mA); (4) the effect of interstimulation interval (groups 3 vs. 6, 1 s vs. 10 s); (5) same total charge with different total energy (groups 3 vs. 8); (6) same total energy with different total charge (groups 3 vs. 7); and (7) the overall association between total charge or energy and lesion depth across all groups. Full stimulation parameters for each group are provided in Table 1.

Total stimulation charge (Q) was defined as Q = I × t, where I is the stimulation current, and t is the total stimulation time (pulse duration × number of stimulations). Stimulation energy was expressed as proportional to I^2^t (W ∝ I^2^t), assuming relatively constant tissue resistance across experimental conditions; accordingly, relative differences in I²t were used as a surrogate for energy comparison across groups. Because a single electrode geometry (2 mm diameter) with constant surface area was used throughout, charge density per phase co-varied exactly with total charge; electrode surface area was therefore held constant rather than treated as an independent variable—a deliberate design choice intended to isolate the contributions of total charge and total energy while minimizing the number of animals in accordance with the 3R principles.

The platinum electrodes were removed immediately after stimulation. Animals were humanely euthanized by an overdose of pentobarbital (150 mg/kg, intraperitoneally) in accordance with institutional and international guidelines. Whole-body perfusion fixation with 9% formaldehyde was performed, and then the removed brain was formalin-fixed. After appropriate fixation, the brain was vertically and coronally sectioned at the center position of the anodal electrode and then paraffin-fixed. A brain preparation was created with a thickness of 3.0 μm from the stimulated brain surface and stained with hematoxylin and eosin. Under an optical microscope, the maximum depth of the lesion in the cerebral cortex of the anodal stimulation was measured.

The depth of the cerebral cortex lesion between the groups of rats was compared using the Mann-Whitney and Kruskal-Wallis tests. The correlation between stimulation charge and energy with the lesion depth was statistically analyzed with multiple linear regression. The p-value was considered significant if *p* < 0.05. Statistical analysis was performed using SPSS version 21.

## Results

**Fig. 2:**
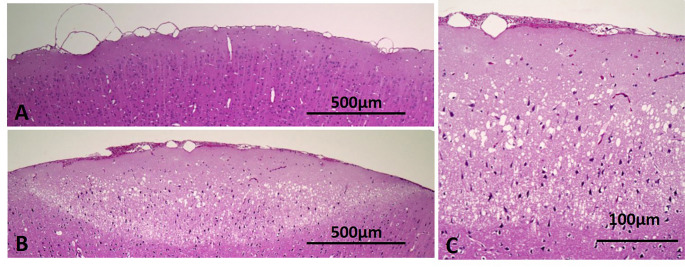
Light microscopic examination did not show any histological changes in the brain tissue of the rats from groups 1 (control) and 2 (**A**), while a downwardly directed convex hemispherical lesion was observed from the surface to the deeper part of the cortex in rats in groups 3 to 8 (**B**). Magnified photo confirming features such as tissue vacuolation, swelling, and microbleeding (**C**)

Transcortical stimulation was delivered to all groups except the control (group 1) according to the research protocol. Electrode–tissue impedance during stimulation was approximately 1 kΩ, as monitored by the constant-current stimulator; per-animal impedance was not systematically logged. Because a constant-current source was used, the delivered current—and thus total charge—was independent of moderate impedance variation, whereas the actual power dissipated (P = I^2^R) depends on tissue resistance. Stimulation energy was therefore analyzed only as a relative index (see Limitations). The brain tissues under the anodal electrode were observed under an optical microscope. There were no histological changes found in rats in groups 1 (control) and 2 (Fig. 2A). A downwardly directed convex hemispherical lesion was observed from the surface of the cortex to a deeper part of the cortex, consistent with the part of the electrode in contact with the disc plane in the rats in groups 3–8 (Fig. 2B). Tissue vacuolation, swelling, and microbleeding were confirmed inside the lesions (Fig. 2C). Qualitatively, these changes were concentrated in the superficial cortex directly beneath the electrode contact and became less marked toward the lateral and deep margins of the hemispherical lesion.

The lesion depth, which was the distance between the cortical surface and the deepest part of the lesion, was measured. The lesion depth ranged from 0 μm to 296 μm, with an average of 122.8 ± 101.3 μm. The average lesion depth for each group with their respective parameters of electrical stimulation is shown in Table 1. The main findings are summarized below.

Regarding stimulation number, lesion depth increased significantly with greater repetition: groups 1 and 2 showed no lesion (0 μm), whereas group 3 reached 209 ± 67 μm (*p* = 0.005 for groups 1–3), and group 7 reached 211 ± 59 μm compared with 97 ± 33 μm in group 4 (*p* = 0.02). Higher stimulation current also produced significantly greater lesion depth: 209 ± 67 μm (group 3, 50 mA) vs. 97 ± 33 μm (group 4, 20 mA; *p* = 0.04). All comparisons are summarized in Table 2.

**Table 2:** Comparison of different stimulation parameters toward lesion depth

Compared parameters	Shared parameters	Total charge (µC)	Rat group	Mean lesion depth (µm)	*p* Value
Stimulation number 1 (times)	SC:50 mA, SD: 0.2 ms, SI: 1s				0.005
0x		0	Group 1	0	
10x		500	Group 2	0	
100x		5000	Group 3	209 ± 67	
Stimulation number 2 (times)	SC:20 mA, SD: 0.2 ms, SI: 1s				0.02
100x		2000	Group 4	97 ± 33	
625x		12,500	Group 7	211 ± 59	
Stimulation current (mA)	SD: 0.2 ms, SN:100x, SI: 1s				0.04
20 mA		2000	Group 4	97 ± 33	
50 mA		5000	Group 3	209 ± 67	
Stimulation duration 1 (ms)	SC: 50 mA, SN: 100x, SI: 1s				0.56
0.05 ms		1250	Group 5	157 ± 141	
0.2 ms		5000	Group 3	209 ± 67	
Stimulation duration 2 (ms)	SC: 20 mA, SN: 100x, SI: 1s				0.25
0.2 ms		2000	Group 4	97 ± 33	
0.5 ms		5000	Group 8	125 ± 101	
Stimulation interval (s)	SC: 50 mA, SD: 0.2 ms, SN: 100x				0.09
1s		5000	Group 3	209 ± 67	
10s		5000	Group 6	183 ± 36	

SC: stimulation current, SD: stimulation duration, SI: stimulation interval, SN: stimulation number

In contrast, neither pulse duration nor interstimulation interval significantly affected lesion depth. Increasing duration from 0.05 to 0.2 ms (groups 5 vs. 3: 157 ± 141 vs. 209 ± 67 μm, *p* = 0.56) and from 0.2 to 0.5 ms (groups 4 vs. 8: 97 ± 33 vs. 125 ± 101 μm, *p* = 0.25) produced numerically larger lesions, but the differences did not reach significance. Similarly, extending the interstimulation interval from 1 to 10 s (groups 3 vs. 6: 209 ± 67 vs. 183 ± 36 μm, *p* = 0.09) showed a trend toward smaller lesions without reaching significance (Table 2).

**Fig. 3:**
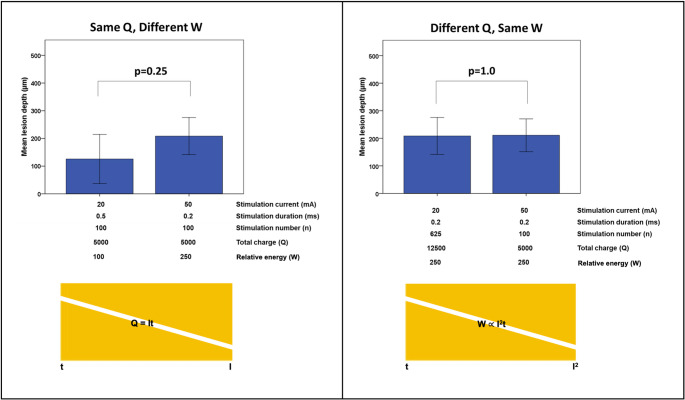
A comparison of lesion depth between rats stimulated with the same total stimulation charge but different stimulation energies in rats in groups 3 and 8 (left) and stimulated with the same total stimulation energy but different stimulation charges in rats in groups 3 and 7 (right). Although the differences did not reach statistical significance, lesion depth was consistently greater in the higher-energy condition, whereas no detectable difference was observed when total energy was equivalent (*p* = 0.25 and *p* = 1.0, respectively). Error bars represent ± standard deviation

**Fig. 4:**
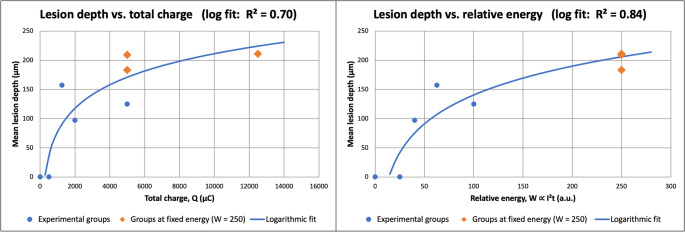
Univariate regressions of maximum cortical lesion depth across all eight groups against total charge (left) and relative stimulation energy (right). In both cases the relationship was better described by a logarithmic than by a linear fit, with relative stimulation energy providing the stronger fit (logarithmic R^2^ = 0.84 for energy versus 0.70 for charge). Diamond markers indicate the three groups sharing identical relative energy (W = 250 arbitrary units; Groups 3, 6, and 7). Despite substantial differences in total charge, these groups exhibited similar lesion depths, consistent with the view that lesion depth is more closely related to stimulation energy than to charge alone

When total charge was held constant, but total energy differed (groups 3 vs. 8: 5,000 µC for both; relative energy I²t: 250 vs. 100 [arbitrary units]), mean lesion depth was numerically greater in the higher-energy group (209 ± 67 vs. 125 ± 101 μm, *p* = 0.25; Fig. 3, left). Conversely, when total energy was equalized but total charge differed 2.5-fold (groups 3 vs. 7: 5,000 vs. 12,500 µC; relative energy I²t: both 250 [arbitrary units]), no detectable difference in lesion depth was observed (209 ± 67 vs. 211 ± 59 μm, *p* = 1.0; Fig. 3, right). These two matched-pair comparisons, which dissociate total charge from total energy, are summarized in Table 3. Multiple linear regression analysis across all groups yielded the equation: lesion depth = 39.24 + 0.02 × Q + 0.61 × W. Total stimulation energy was the only significant independent predictor (*p* = 0.008), whereas total stimulation charge was not (*p* = 0.677). Notably, total charge and relative energy were themselves strongly correlated across the experimental groups (Pearson *r* = 0.80); consequently, neither the pooled correlation nor the multiple regression can by itself fully separate their individual contributions, and the matched-charge and matched-energy comparisons (Groups 3 vs. 7 and Groups 3 vs. 8) provide the principal basis for dissociating the two parameters. Univariate regressions of lesion depth on charge and on energy across all groups were each better described by a logarithmic than by a linear function, with energy providing the closer fit (logarithmic R^2^= 0.84 for energy vs. 0.70 for charge; Fig. 4). The maximum lesion depth of all eight groups is presented individually in Table 1 and plotted by group in Fig. 4.

**Table 3:** Dissociation of total charge and total energy as determinants of lesion depth in matched-pair comparisons

Compared parameter	Shared parameter	Total charge (µC)	Relative energy (I^2^t)	Rat group	Mean lesion depth (µm)	*p* Value
Total energy	Total charge = 5000 µC					0.25
		5000	100	Group 8	125 ± 101	
		5000	250	Group 3	209 ± 67	
Total charge	Relative energy (I^2^t) = 250					1.0
		5000	250	Group 3	209 ± 67	
		12,500	250	Group 7	211 ± 59	

In each comparison one quantity was held constant while the other was varied. *p* values (Mann–Whitney) are reported for completeness; given the small group size (*n* = 4), non-significant values should not be interpreted as evidence of equivalence

## Discussion

The present study examined the relative biophysical contributions of electrical charge and stimulation energy to cortical lesion depth under MEP-relevant high-frequency stimulation. Stimulation current and the number of stimulations were the parameters most consistently associated with greater lesion depth. Most importantly, controlled comparisons between groups with equivalent total charge but differing total energy suggested that parameters contributing to total stimulation energy (W ∝ I^2^t) may play a greater role in determining lesion depth than total charge (Q = I × t) alone, a pattern further supported by multiple regression in which total energy was the only significant independent predictor (*p* = 0.008) while total charge was not (*p* = 0.677). Although the small group sizes (*n* = 4 per group) limited statistical power for detecting subtle effects of pulse duration or interstimulation interval, the consistency of the energy-dependent pattern across multiple controlled comparisons supports the validity of this mechanistic framework and indicates that energy deposition may be an important biophysical driver of cortical injury under these conditions.

Electrical tissue damage beneath a stimulating electrode is histologically characterized by neuronal vacuolation, edema, disruption of cytoarchitecture, vasodilation, and microhemorrhage (Yuen et al. 1981; McCreery et al. 1990). Multiple physicochemical mechanisms have been implicated, including gas evolution, local pH changes, resistive heating, and direct electrochemical effects on cellular membranes (MacDonald 2002; Berendson and Simonsson 1994; Butterwick et al. 2007). These factors accumulate with increasing stimulation intensity (Yuen et al. 1981; Berendson and Simonsson 1994). At the anode in particular, oxidation reactions produce local acidification, which is considered a primary driver of tissue injury (Berendson and Simonsson 1994). The ohmic voltage drop across the electrode–tissue interface contributes relatively little to tissue damage, as most of the applied charge is consumed in charging the electrical double layer rather than driving faradaic reactions. Collectively, these mechanisms converge through tissue electrolysis to produce the observed lesion pattern.

Prior work on chronic cortical stimulation has emphasized charge density per phase and total charge density as key determinants of neural injury, with McCreery et al. demonstrating a synergistic relationship between charge density and charge per pulse (Yuen et al. 1981; McCreery et al. 1990). Shannon’s model similarly highlighted charge density as a primary predictor of damage thresholds (Cogan et al. 2016; Yuen et al. 1981; McCreery et al. 1990; Gordon et al. 1990; MacDonald 2002; Shannon 1992). However, those safety criteria were derived from chronic, low-frequency paradigms and may not directly translate to the brief, high-frequency, repetitive stimulation used for intraoperative MEP monitoring (Pechstein et al. 1996; Oinuma et al. 2007). Furthermore, inconsistencies in the relationship between charge density and neural damage across prior studies suggest that additional factors beyond charge alone may contribute to electrical injury during MEP-relevant stimulation.

## Energy versus charge as determinants of tissue injury

The present results suggest that, under MEP-relevant stimulation conditions, total energy may be a more informative predictor of lesion depth than total charge. In the key controlled comparison with matched total charge (Groups 3 vs. 8), the higher-energy group showed a numerically greater mean lesion depth, whereas equalizing total energy despite a 2.5-fold difference in total charge (Groups 3 vs. 7) produced no detectable difference (Table 3). Although neither comparison reached statistical significance (likely reflecting the limited sample size), the consistent directional pattern across both experiments supports the hypothesis that energy-related parameters may contribute more strongly to lesion depth than charge-related parameters under these conditions. Because total charge and total energy were strongly correlated across groups, it is this experimental dissociation—rather than the pooled correlation of either parameter with lesion depth—that provides the principal evidence that energy-related parameters contribute more to lesion depth than charge alone. Across all groups, lesion depth rose with charge and with energy in a saturating, logarithmic rather than linear manner (Fig. 4). A plausible reading is a ceiling effect: once the superficial cortex immediately beneath the contact is maximally injured, further charge or energy deepens the lesion with diminishing returns, and the closer fit for energy is consistent with energy deposition being the more proximate determinant of depth.

## Biophysical mechanisms: resistive heating and electrolysis

A plausible mechanistic basis for this energy dependence lies in resistive heating. Because power dissipation in tissue scales with the square of current (P = I²R), a higher stimulation current generates disproportionately more heat per unit time than a lower current delivering equivalent charge over a longer pulse duration. This I^2^ dependence of energy deposition may explain why stimulation current emerged as a significant predictor of lesion depth, while pulse duration—despite influencing total charge—did not. The pulse durations tested in the present study (0.05–0.5 ms) span a range that overlaps with the chronaxie of cortical gray matter (0.2–0.7 ms). Charge delivery near chronaxie is considered physiologically efficient for neural activation (Ranck 1975; Abalkhail et al. 2017), but this same range may limit the independent contribution of pulse duration to electrochemical injury when current is held constant. The present results are consistent with this interpretation: in comparisons where total charge was equalized between groups but current differed (Groups 4 vs. 3 and Groups 4 vs. 8), lesion depth was consistently greater in the higher-current group, further suggesting that current magnitude—and by extension, energy—may be more relevant than pulse duration to the depth of cortical injury.

The present findings may also help reconcile apparent discrepancies between our results and prior charge-density-based models (Cogan et al. 2016; Yuen et al. 1981; McCreery et al. 1990). Charge density tends to be highest at the electrode periphery, which may contribute predominantly to surface morphological damage rather than to the depth of injury measured here. Stimulation energy, by contrast, may better reflect the volumetric extent of tissue heating and electrolytic injury beneath the electrode. This distinction—charge density as a predictor of surface morphological severity versus energy as a predictor of lesion depth or volume—may help reconcile apparent discrepancies between prior histological studies and the present depth-based measurements.

## Implications for safer stimulation protocols

These findings carry several practical implications for the safe conduct of intraoperative MEP monitoring, bearing in mind their exploratory nature. When it is necessary to increase stimulation intensity—for example, in patients with preoperative motor deficits, high stimulation thresholds, or monitoring of muscles with inherently high thresholds such as lower extremity musculature (Hardian et al. 2021)—the present data suggest that increasing pulse duration rather than stimulation current may, as a hypothesis-generating consideration requiring direct validation, be the safer strategy, as it increases total charge with a smaller proportional increase in energy. Keeping the pulse duration near the chronaxie of the target tissue would be expected to maintain stimulation efficiency while minimizing energy deposition (Abalkhail et al. 2017). Second, the strong influence of stimulation number on lesion depth underscores the importance of threshold-based stimulation protocols that minimize unnecessary repetitions (Abboud et al. 2016; Calancie et al. 2001; Hardian et al. 2019). The threshold was defined in the present study as the lowest intensity yielding a reproducible MEP response with amplitude ≥ 20 µV and appropriate latency (Hardian et al. 2021; Goto et al. 2010; Kanaya et al. 2019a), an approach that limits cumulative energy deposition while preserving monitoring sensitivity. When no MEP response is obtained, the stimulation number must be increased, though a certain additional margin may be acceptable to ensure reliable clinical monitoring.

It is important to note that the stimulation parameters and electrode geometry used here are not directly representative of clinical practice. Standard intraoperative transcortical electrodes have a diameter of approximately 5 mm, whereas the 2 mm electrodes used in the present study are smaller. For equivalent stimulation current (20 mA), the charge density per phase beneath a 5 mm electrode is approximately 1/15 that of a 2 mm electrode, and if tissue change scales proportionally with energy, the effective damage would be expected to be substantially reduced (Goto et al. 2018; Kanaya et al. 2019b). Furthermore, in clinical practice, stimulation is typically delivered at intervals of several seconds to minutes rather than the 1-second interval used here, allowing greater thermal and electrochemical dissipation between trials. These differences suggest that tissue injury under typical clinical conditions would be substantially lower than that observed experimentally, consistent with the overall clinical safety record of transcortical MEP monitoring. Accordingly, the present findings should be interpreted as providing a mechanistic framework rather than a direct representation of clinical conditions, and caution should be exercised when extrapolating to the clinical setting (Taniguchi et al. 1993; Neuloh and Schramm 2009). Nevertheless, the present results serve as a reminder that apparently safe stimulation intensities may produce histological changes at the site of electrode contact, particularly under conditions of prolonged surgery or a high number of stimulation trials, and that minimizing total stimulation energy throughout the procedure remains prudent.

### Study limitations and exploratory nature

Several limitations must be acknowledged. First, rat and human cortex differ in geometric organization and cellular density, and the tolerance of human cortex to electrical stimulation may not be identical to that of the rat (Hodge et al. 2019; DeFelipe 2011). Nevertheless, the fundamental cytoarchitecture and cellular constituents of the mammalian cerebral cortex are broadly conserved, supporting the relevance of the rat model for mechanistic investigation. Second, the present study measured lesion depth as a surrogate for injury severity. Whether the observed histological changes translate into functional motor deficits remains unknown and warrants dedicated investigation. In addition, because lesion depth was prospectively defined as the primary endpoint, systematic semi-quantitative scoring of neuronal vacuolation, edema, vasodilation, and microhemorrhage was not undertaken; these features were characterized qualitatively in the present study, and their formal quantification remains an objective for future work. Third, only a single electrode size was employed, precluding direct assessment of how electrode surface area modulates the relationship between energy, charge density, and injury depth. Variability in electrode contact area—due to intraoperative handling or gas formation at the electrode–tissue interface—represents an additional source of uncertainty in both experimental and clinical settings. Fourth, the small group size (*n* = 4 per group) substantially limits statistical power and generalizability. The non-significant p-values observed in several comparisons should therefore be interpreted with caution; they may reflect insufficient power to detect true differences rather than the absence of an effect. Replication with larger cohorts and a broader range of stimulation parameters is needed to confirm these observations. Finally, because brain tissue is electrically inhomogeneous and anisotropic (Aström et al. 2012), the simplified assumption of constant resistance—including inter-animal and inter-electrode variation in electrode–tissue impedance—used to calculate energy may introduce systematic error. In this study, stimulation energy was treated as a relative index proportional to I^2^t, rather than an absolute quantity, because tissue resistance was not directly measured. Accordingly, the energy values reported here should be interpreted as relative comparisons between groups rather than true physical energy values. More refined models incorporating direct tissue impedance measurements would be required to validate this approach and strengthen future analyses. Importantly, although the dissociation comparisons were designed to partially separate the contributions of charge and energy, the present study was not specifically powered as a confirmatory experiment to establish energy dependency; dedicated studies with larger cohorts designed for that purpose will be required, and the absence of such a confirmatory experiment should be regarded as a principal limitation of this work.

## Conclusions

Under high-frequency transcortical stimulation conditions, the depth of cortical injury appears to be more closely associated with total stimulation energy (W ∝ I^2^t) than with total electrical charge alone, consistent with biophysical mechanisms involving resistive heating and electrolytic processes at the electrode–tissue interface. Stimulation current and the number of stimulations appear to be the parameters most strongly associated with lesion depth, whereas no significant independent effects of pulse duration and interstimulation interval were detected within the parameter ranges examined. These findings provide a mechanistic framework that may inform safer stimulation protocols: when stronger stimulation output is required, increasing pulse duration at constant current may be preferable to increasing current itself, as it raises total charge with a smaller proportional rise in energy. Replication in larger cohorts and with direct tissue impedance measurements is needed to establish quantitative thresholds across the parameter space.

## Data Availability

No datasets were generated or analysed during the current study.
